# Health-Related Quality of Life and Symptom Burden in Patients with Diffuse Large B-Cell Lymphoma Before Treatment with Tafasitamab and Lenalidomide: An Ad Hoc Analysis of Italian Real-World Data from the PRO-MIND Study

**DOI:** 10.3390/diseases13120399

**Published:** 2025-12-15

**Authors:** Pier Luigi Zinzani, Nicola Battaglia, Mario Lapecorella, Guido Gini, Maria Cristina Cox, Stefan Hohaus, Antonio Pinto

**Affiliations:** 1 Institute of Hematology, IRCCS Azienda Ospedaliero-Universitaria di Bologna, Department of Medical and Surgical Sciences, Bologna University School of Medicine, 40138 Bologna, Italy; pierluigi.zinzani@unibo.it; 2 Incyte Biosciences Italy SRL, 20124 Milano, Italy; mlapecorella@incyte.com; 3 Divisione di Ematologia, Azienda Ospedaliera Universitaria Ospedali Riuniti, 60126 Ancona, Italy; guido.gini@ospedaliriuniti.marche.it; 4 UOC Malattie Linfoproliferative, Fondazione Policlinico Tor Vergata, 00133 Rome, Italy; mariacristina.cox@ptvonline.it; 5 Department of Radiological and Hematological Sciences, Fondazione Policlinico Universitario A. Gemelli IRCCS, Università Cattolica del Sacro Cuore, 00168 Rome, Italy; stefan.hohaus@unicatt.it; 6 Hematology-Oncology and Stem Cell Transplantation Unit, Istituto Nazionale Tumori, Fondazione ‘G. Pascale’, IRCCS, 80131 Naples, Italy; a.pinto@istitutotumori.na.it

**Keywords:** diffuse large B-cell lymphoma, relapsed/refractory, health-related quality of life, symptom burden, tafasitamab, lenalidomide, real-world data

## Abstract

**Background/Objectives**: Diffuse large B-cell lymphoma (DLBCL) exhibits substantial clinical heterogeneity and poor prognosis in relapsed/refractory (R/R) settings. PRO-MIND is a prospective, multicenter real-world study evaluating tafasitamab–lenalidomide followed by tafasitamab monotherapy in patients with transplant-ineligible R/R DLBCL in Italy. This ad hoc, cross-sectional, baseline analysis aimed to characterize health-related quality of life (HRQoL) and symptom burden before tafasitamab–lenalidomide initiation in the PRO-MIND cohort. **Methods**: Thirty-eight patients across 30 centers completed the EORTC QLQ-C30 and QLQ-NHL-HG29 questionnaires at pretreatment baseline, prior to starting tafasitamab–lenalidomide. EORTC QLQ-C30 scores (0–100) were compared with age-specific normative values for the Italian general population using Welch’s t-test. Differences of ≥5 points were considered clinically meaningful and ≥10 points clearly clinically important. Effect sizes (Cohen’s d) were calculated to complement *p*-values for between-group comparisons. **Results**: Compared with normative data, the PRO-MIND cohort had significantly lower EORTC QLQ-C30 functioning scores for physical (Δ 12.7, *p* = 0.0135), role (Δ 16.1, *p* = 0.0168), social (Δ 15.2, *p* = 0.0019), and cognitive (Δ 8.5, *p* = 0.0460) functioning. Symptom scales revealed worse fatigue (Δ 14.8, *p* = 0.0097), insomnia (Δ 13.9, *p* = 0.0291), appetite loss (Δ 9.4, *p* = 0.0435), and pain (Δ 8.7, *p* = 0.0430) in the PRO-MIND cohort versus normative data, with effect sizes in the small-to-moderate range. EORTC QLQ-NHL-HG29 scores indicated a high prevalence of concerns about future health (84.2%), disease recurrence (81.6%), and dependency (78.9%), as well as physical symptoms, including lack of energy (71.1%), sleep difficulties (63.2%), and pain or discomfort (60.5%). **Conclusions**: This cross-sectional, baseline-only analysis of the PRO-MIND real-world cohort showed that patients with transplant-ineligible R/R DLBCL scheduled to receive tafasitamab–lenalidomide already had pronounced impairments in physical, role, social, and cognitive functioning, along with substantial fatigue, insomnia, pain, appetite loss, and psychological concerns. These baseline benchmarks underscore the importance of systematic HRQoL assessment and targeted supportive interventions focusing on these domains before and during treatment. Future longitudinal PRO-MIND analyses will complement these findings by describing how HRQoL evolves after tafasitamab–lenalidomide initiation.

## Simple Summary

Patients with relapsed or refractory diffuse large B-cell lymphoma often experience severe symptoms and a reduced quality of life, yet little is known about their well-being before starting new therapies. In this study, we collected real-world data on how patients with relapsed or refractory diffuse large B-cell lymphoma feel and function before starting treatment with a combination of tafasitamab and lenalidomide. By comparing these experiences to those of healthy individuals of the same age, we identified key areas—such as fatigue, pain, and concerns about future health and disease recurrence—where patients with relapsed or refractory diffuse large B-cell lymphoma struggle most. These insights will help researchers design better supportive care strategies and more accurately evaluate the real impact of emerging treatments on the daily lives of these patients.

## 1. Introduction

Diffuse large B-cell lymphoma (DLBCL) is the most common non-Hodgkin lymphoma in adults, and up to 40% of patients develop relapsed or refractory (R/R) disease, particularly older and comorbid individuals who are not candidates for intensive salvage chemotherapy or autologous stem-cell transplantation [1,2]. Tafasitamab, an Fc-engineered anti-CD19 monoclonal antibody, in combination with lenalidomide (tafasitamab–lenalidomide) followed by tafasitamab monotherapy, has shown durable responses and encouraging survival in patients with transplant-ineligible R/R DLBCL in clinical trials and real-world cohorts [3,4]. In routine practice, this regimen is often selected for older or frailer patients who are not candidates for more intensive second-line options [3,4]. In L-MIND, the regimen was generally well tolerated, with predominantly hematologic adverse events (neutropenia, thrombocytopenia, anemia) and infections, and manageable non-hematologic events such as fatigue and diarrhea, a safety profile broadly confirmed in real-world studies [4,5]. These toxicities, although manageable, may still affect patients’ perceived health and functioning over time.

Patient-reported outcomes (PROs), particularly health-related quality of life (HRQoL), complement traditional endpoints by capturing functional, symptomatic, and psychosocial burden in R/R DLBCL [6]. Emerging PRO data from trials of CAR-T therapy and bispecific antibodies suggest that some patients experience HRQoL preservation or improvement despite treatment-related toxicities [7,8,9]. However, these studies involve highly selected populations and provide little information on pretreatment HRQoL in older, comorbid patients receiving antibody-based therapies, such as tafasitamab–lenalidomide, in routine practice. To date, no detailed description of baseline HRQoL immediately before tafasitamab–lenalidomide initiation in real-world settings has been reported.

PRO-MIND is a prospective, multicenter real-world study in Italian hematology centers evaluating tafasitamab–lenalidomide followed by tafasitamab monotherapy in patients with transplant-ineligible R/R DLBCL. Because baseline HRQoL is prognostic for survival and essential for interpreting subsequent treatment-related changes, there is a need to benchmark HRQoL and symptom burden at the time of the treatment decision in this frail population [10,11]. This ad hoc, cross-sectional, baseline analysis of PRO-MIND aimed to characterize pretreatment HRQoL and symptom burden in patients scheduled to receive tafasitamab–lenalidomide and to compare their European Organization for Research and Treatment of Cancer (EORTC) Quality of Life Questionnaire (QLQ)-Core 30 (C30) scores with age-specific normative values for the Italian general population.

## 2. Methods

The PRO-MIND study began in November 2023 and involves 30 Italian centers. This ad hoc, cross-sectional analysis included all patients enrolled in PRO-MIND up to 16 August 2024, and was restricted to baseline PRO data. This analysis was not pre-specified in the original PRO-MIND protocol but was planned before database lock and execution of the current statistical analyses.

### 2.1. Patients

Patients aged 18 years or older with R/R DLBCL who were not eligible for autologous stem-cell transplantation were included in the study. Patients initiated treatment with commercially available tafasitamab–lenalidomide after signing the informed consent form, with the decision to prescribe tafasitamab made independently of study enrollment. Exclusion criteria included concurrent participation in an interventional clinical trial, patients whose initial diagnosis or follow-up data were deemed unreliable for the observational purposes of the study (according to the physician’s judgment), and patients who had begun tafasitamab before signing the informed consent form.

### 2.2. Assessments

Demographics and clinical characteristics, in addition to PROs, were collected at baseline, prior to the initiation of tafasitamab–lenalidomide. HRQoL was assessed using the Italian version of the EORTC QLQ-C30 [12], and the EORTC Quality of Life Questionnaire for High-Grade Non-Hodgkin Lymphoma (EORTC QLQ-NHL-HG29) to capture lymphoma-specific concerns [13].

The questionnaires were primarily administered electronically (ePROs) to ensure standardized data collection. When electronic completion was not feasible, patients received a paper version [14]. All questionnaires were administered at the hospital immediately before the clinical visit and completed by patients independently, with only minimal assistance from study staff to avoid response bias [15]. Questionnaires were completed in a quiet area of the clinic, without time limits. Study staff could provide technical assistance only (e.g., reading questions aloud for visually impaired patients or clarifying response options) but were instructed not to suggest or influence answers. All responses were recorded directly by patients. All baseline HRQoL and symptom burden data were collected prior to treatment initiation to establish a pretreatment reference point.

Compliance was actively monitored, and in cases of missing data, a missing data form was required from each participating center. For several EORTC QLQ-NHL-HG29 items that are less applicable to older, retired individuals (e.g., problems at work/place of study, worries about work/study, concerns about ability to have children), item-level missingness was high (approximately 50%), and these items were analyzed descriptively only. In this elderly, predominantly retired cohort, problems at work or place of study were interpreted as reflecting limitations in role functioning and usual daily activities rather than paid employment or formal education. No formal comparative analyses were performed between ePRO and paper completers, and digital literacy was not systematically assessed.

### 2.3. Statistical Analyses

Scores for the EORTC QLQ-C30 and QLQ-NHL-HG29 were transformed to a 0–100 scale, with higher scores indicating better function on functioning scales and greater symptom burden on symptom scales [16]. Descriptive statistics were used to summarize patient characteristics and HRQoL scores. Continuous variables were reported as mean, standard deviation, median, interquartile range (IQR), and minimum–maximum values, while categorical variables were reported as frequency counts and percentages. Missing data were not imputed; only non-missing items were used to calculate scale scores according to the EORTC scoring manuals, and only non-missing observations were included in the analyses. The EORTC QLQ-NHL-HG29 data were handled descriptively: items rated “a little”, “quite a bit” or “very much” were considered positive, and the resulting proportion of symptomatic patients was calculated in the absence of specific comparative benchmarks [17].

QLQ-C30 scores from the PRO-MIND cohort were compared with age-specific normative values for the Italian general population reported in the literature [18]. Normative data were obtained from an Italian subsample of an international EORTC project collected via online panels (March–April 2017) using quota sampling by sex and age group (18–39, 40–49, 50–59, 60–69, and ≥70 years), with post-stratification weights applied to match the UN age- and sex-distribution of the Italian population. The resulting weighted sample comprised 1036 adults (mean age 49.3 years; 51.7% female) and provided estimates stratified by sex, age group, and health condition status (none vs. ≥1 doctor-diagnosed condition) [18]. Given the large normative dataset and the possibility of unequal variances between groups, Welch’s *t*-test for independent samples was used to assess differences in mean scores for each scale. To complement *p*-values, effect sizes (Cohen’s d) were calculated for each comparison.

Because this ad hoc baseline analysis is exploratory and involves multiple HRQoL domains, no formal adjustment for multiple testing was performed; all *p*-values are therefore descriptive and should be interpreted with caution. Differences of ≥5 points were considered clinically meaningful and ≥10 points as clearly clinically important, in line with EORTC guidance [19]. Given the small sample size (N = 38), the observational design, and the susceptibility of PROs to response bias, no multivariable models were fitted, and all findings should be regarded as hypothesis-generating. In particular, we did not perform formal multivariable or extensive univariable correlations between HRQoL and clinical or biological factors (e.g., disease bulk, Eastern Cooperative Oncology Group performance status [ECOG PS], refractory versus relapsed disease, inflammatory markers, number of prior therapies) because of limited power.

## 3. Results

### 3.1. Baseline Characteristics

A total of 38 patients were included in the analysis, with a median age of 77 years (IQR: 73.0–82.0); 23 (60.5%) were female and 37 (97.4%) were White (Table 1). At baseline, 35 (92.1%) patients had ≥1 preexisting comorbidity, most commonly hypertension (*n* = 23; 60.5%), hypercholesterolemia (*n* = 6; 15.8%), diabetes (*n* = 5; 13.2%), depression (*n* = 5; 13.2%), thyroid disorder (*n* = 4; 10.5%), and atrial fibrillation (*n* = 4; 10.5%)**.** Overall, 22/37 (59.5%), 8/37 (21.6%), and 7/37 (18.9%) patients had Ann Arbor stages IV, III, and II disease, respectively, and 31/37 (83.8%) had DLBCL not otherwise specified. Prior to enrollment, 15/37 (40.5%), 17/37 (45.9%), and 5/37 (13.5%) patients achieved a complete response, partial response, and stable disease following first-line therapy, respectively. ECOG PS was recorded for 30 (78.9%) patients; among these, 14 (46.7%), 15 (50.0%), and 1 (3.3%) had scores of 0, 1, and 2, respectively.

### 3.2. Prior Treatments

Of the 37 patients with available data, 22 (59.5%) had received 1 prior systemic regimen, 12 (32.4%) had undergone 2 prior regimens, and 3 (8.1%) had received 3 regimens (Table 1). Nearly all patients (34/37; 91.9%) had received a CD20-targeted therapy, and the majority (26/37; 70.3%) had completed 6 cycles of treatment.

Among patients with available information (*n* = 37), prior therapy was most commonly rituximab-cyclophosphamide-oncovin-non-pegylated liposomal doxorubicin-prednisone (R-COMP)-based regimens (22/37, 59.5%), followed by rituximab-cyclophosphamide-hydroxydaunorubicin-oncovin-prednisone (R-CHOP)–based treatment (including attenuated R-mini-CHOP) (5/37, 13.5%), polatuzumab-based combinations (e.g., polatuzumab-rituximab-cyclophosphamide-doxorubicin-prednisone or rituximab–polatuzumab ± bendamustine) (5/37, 13.5%), and other regimens (dose adjusted etoposide phosphate-prednisone-oncovin-cyclophosphamide-doxorubicin-rituximab/rituximab-etoposide phosphate-prednisone-oncovin-cyclophosphamide-doxorubicin, rituximab-gemcitabine-dexamethasone-platinum ± selinexor, rituximab-gemcitabine-oxaliplatin, and cyclophosphamide-mitoxantrone-vincristine-etoposide-bleomycin-prednisone) (5/37, 13.5%). Overall, most patients had received CHOP-like chemoimmunotherapy, frequently with R-COMP in the prior-treatment history.

As second-line treatment, 4 patients (33.3%) received a polatuzumab-based salvage regimen, predominantly polatuzumab-bendamustine-rituximab or other polatuzumab-containing combinations. Other commonly used second-line regimens included rituximab-gemcitabine-dexamethasone-platinum (*n* = 1; 8.3%), rituximab-gemcitabine-oxaliplatin (*n* = 1; 8.3%), dose-adjusted R-EPOCH (*n* = 1 8.3%), and vincristine-mitoxantrone-cyclophosphamide-oncovin-prednisone-bleomycin (*n* = 1; 8.3%). A subset of patients (*n* = 4; 33.3%) received modified R-COMP or rituximab-based regimens in combination with other agents. Additionally, 4 patients (10.8%) underwent radiotherapy as part of their prior treatment history, all of whom completed the planned treatment course.

### 3.3. HRQoL and Symptom Burden

**Figure 1:**
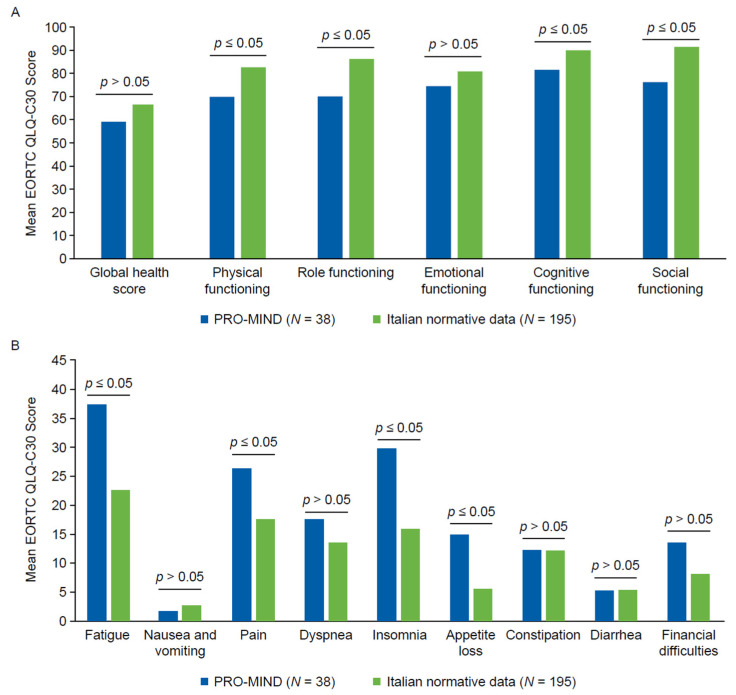
Health-related quality of life in the PRO-MIND cohort versus age-specific normative values for the Italian general population. (**A**) Mean EORTC QLQ-C30 scores for the global health score and the five functioning scales. Higher scores indicate better quality of life. Significant impairments in physical, role, cognitive, and social functioning were reported for the PRO-MIND cohort versus normative values. (**B**) Mean EORTC QLQ-C30 scores for the eight symptom scales. Higher scores denote greater symptom burden. Patients in the PRO-MIND cohort reported significantly greater fatigue, pain, insomnia, and appetite loss versus normative values. Abbreviations: C30, Core 30; EORTC, European Organization for Research and Treatment of Cancer; QLQ, Quality of Life Questionnaire.

Of the 38 patients included in this ad hoc baseline analysis, all 38 (100%) completed the EORTC QLQ-C30, and 36 (94.7%) completed the EORTC QLQ-NHL-HG29 with sufficient data to analyze the main scales; 2 patients had missing EORTC QLQ-NHL-HG29 questionnaires. For the EORTC QLQ-NHL-HG29, scale-level N values ranged from 36/38 (94.7%) for symptom burden, neuropathy, physical condition/fatigue, emotional impact and worries about health and functioning to 18–19/38 (47–50%) for items related to work/study and fertility, reflecting the limited applicability in this predominantly older cohort. Comparative analysis of EORTC QLQ-C30 scores between patients in the PRO-MIND study and age-specific normative values for the Italian general population revealed significant impairments in multiple domains of HRQoL. In terms of the functioning scales, statistically significant reductions in the PRO-MIND cohort were observed for physical functioning (Δ 12.69, *p* = 0.0135), role functioning (Δ 16.14, *p* = 0.0168), social functioning (Δ 15.19, *p* = 0.0019), and cognitive functioning (Δ 8.52, *p* = 0.0460) (Figure 1A).

In terms of symptom scales, statistically significant worsening in the PRO-MIND cohort was observed for fatigue (Δ 14.84, *p* = 0.0097), pain (Δ 8.70, *p* = 0.043), insomnia (Δ 13.89, *p* = 0.0291), and appetite loss (Δ 9.37, *p* = 0.0435) (Figure 1B; Supplementary Table S1). For the domains with ≥10-point differences versus age-specific normative values for the Italian general population (physical, role and social functioning, fatigue, and insomnia), effect sizes were in the moderate-to-large range (Cohen’s d ≈ 0.5–0.9; Supplementary Table S1), supporting the clinical relevance of these impairments.

Analysis of the EORTC QLQ-NHL-HG29 questionnaire responses highlighted the substantial impact of disease and treatment burden on HRQoL in patients with DLBCL enrolled in PRO-MIND (Table 2). The most frequently reported concerns and symptoms revealed a predominantly psychological and physical toll on patients. Specifically, 84.2% of patients expressed worries about their future health, making it the most common reported issue. Similarly, 81.6% of patients reported concerns about disease recurrence, reflecting the persistent psychological distress associated with the risk of relapse. Furthermore, 78.9% of patients worried about becoming dependent on others, emphasizing the fear of losing autonomy and requiring long-term assistance.

Among physical symptoms, 71.1% of patients experienced a lack of energy, indicating a high burden of fatigue. The perception of chronic illness was also a major concern, with 68.4% fearing the possibility of developing a chronic condition. Other highly prevalent symptoms included difficulty sleeping (63.2%), pain or discomfort (60.5%), and problems with memory or concentration (57.9%).

When considering symptoms and concerns present in at least 50% of patients, the dataset further reinforced the widespread impairment in HRQoL. More than half of patients reported difficulties in carrying out daily activities (55.3%), suggesting significant functional limitations. Social restrictions (52.6%) and emotional distress (50.0%) were also frequently reported.

## 4. Discussion

The present cross-sectional baseline analysis of the PRO-MIND cohort shows that patients with transplant-ineligible R/R DLBCL scheduled to receive tafasitamab–lenalidomide already experience marked HRQoL impairments and symptom burden compared with age-specific normative values for the Italian general population. The largest and most consistently clinically meaningful differences were observed in physical, role, and social functioning, with additional deficits in cognitive functioning and prominent symptoms such as fatigue, insomnia, pain, and appetite loss. These findings highlight the substantial multidimensional burden faced by patients at the time of the treatment decision, before any potential benefits or toxicities of tafasitamab–lenalidomide are realized. The pattern of impairment observed in PRO-MIND is likely multifactorial, reflecting the combined influence of advanced age, comorbidities, disease status, and prior therapy, rather than any single determinant. The cohort was characterized by a high median age and a substantial prevalence of vascular, metabolic, and neurological comorbidities, which plausibly contribute to reduced physical and social functioning and to chronic fatigue and pain, as reported in previous lymphoma survivorship studies [20,21,22]. At the same time, the high proportion of patients with advanced-stage and refractory disease and prior exposure to multiple lines of therapy may further exacerbate symptom burden and psychological distress. However, in the absence of multivariable modeling, these relationships remain associative and hypothesis-generating rather than causal. The EORTC QLQ-NHL-HG29 data underscore the substantial symptom burden and psychosocial distress in patients with R/R DLBCL before treatment with tafasitamab–lenalidomide. Over 80% of patients had concerns about disease recurrence (81.6%) and their future health (84.2%), and nearly 80% worried about loss of independence (78.9%), underscoring the long-term psychological consequences of DLBCL. Therefore, while concerns about future health, recurrence and dependency are likely to be influenced by the underlying lymphoma, they may also reflect age-related vulnerabilities and the cumulative burden of comorbid conditions, rather than the disease alone. Fatigue was pervasive (71.1%), which could be related to both disease status and the lasting effects of prior treatment. Sleep difficulties (63.2%), pain or discomfort (60.5%), and problems with memory or concentration (57.9%) were highly prevalent, highlighting the multidimensional nature of disease impact. More than half of patients with R/R DLBCL reported difficulties carrying out daily activities (55.3%) and social restrictions (52.6%), while half experienced emotional distress (50.0%), indicating that the disease experience extended beyond physical symptoms to affect broader aspects of patients’ social interactions and mental well-being. These findings underscore the need for holistic, patient-centered interventions aimed at mitigating both the physical and psychosocial challenges associated with R/R DLBCL [23].

Our results align with previous studies evaluating HRQoL in aggressive B-cell lymphomas prior to intensive therapy. A study demonstrated that patients with R/R high-grade B-cell lymphomas scheduled for CAR-T therapy experienced substantial HRQoL impairments, particularly in role and social functioning, fatigue, dyspnea, and financial difficulties when compared with the general population [24]. Similarly, our findings highlight role and social functioning impairments as major challenges for patients with R/R DLBCL, reinforcing the need for early HRQoL assessment and patient-centered supportive measures before initiating novel therapeutic approaches. Taken together, these observations suggest that the magnitude and pattern of baseline HRQoL impairment in PRO-MIND are broadly comparable to those reported for CAR-T candidates, although direct cross-study comparisons are limited by differences in eligibility criteria, timing of assessments, and PRO instruments. Furthermore, a study of long-term DLBCL survivors emphasized the persistent impact of comorbidities and prior chemotherapy on HRQoL, showing that patients with additional health conditions experienced significantly worse physical functioning, global health status, and emotional distress [25]. Our study extends these findings by demonstrating that patients already have impaired HRQoL, particularly in physical and social domains, before the initiation of second-line treatment, emphasizing the cumulative burden of prior treatment failure.

We employed both the EORTC QLQ-C30 core questionnaire and the disease-specific EORTC QLQ-NHL-HG29 module to optimize our PRO assessment. Normative EORTC QLQ-C30 data for the Italian general population provided reference values for all 15 functioning and symptom scales, which enabled culturally and demographically appropriate benchmarking of our R/R DLBCL cohort (N = 38), though the small sample size limits the precision of such comparisons [18]. To address lymphoma-specific issues, we supplemented the EORTC QLQ-C30 with the EORTC QLQ-NHL-HG29, developed via rigorous, cross-cultural item generation, expert review and pilot testing, and validated in 423 patients with high-grade NHL [13]. Together, these instruments balance broad comparability with targeted sensitivity. In addition, baseline EORTC QLQ-C30 scores have been shown to provide independent prognostic information for survival across multiple tumor types and specifically in DLBCL [10,11], suggesting that the marked deficits observed at baseline in PRO-MIND may also have prognostic relevance, although this cannot be assessed in the current cross-sectional analysis.

This study offers valuable insights into HRQoL prior to the initiation of tafasitamab–lenalidomide; however, several limitations must be acknowledged. The relatively small sample size (N = 38) limits the generalizability of the findings, and larger, multicenter studies are warranted to validate these observations in broader and more diverse R/R DLBCL populations. The study cohort consisted of patients selected based on investigator judgment, potentially introducing selection bias. The cohort’s demographic profile–characterized by a high median age and notable comorbidity burden–likely reflects preferential use of tafasitamab–lenalidomide in frailer patients, suggesting that baseline HRQoL may capture frailty-related impairment as much as disease biology. As a result, HRQoL outcomes reported in this study may not reflect those of patients receiving alternative salvage regimens or palliative care, including polatuzumab-based combinations or CAR-T therapy, as our single-arm design does not include any concurrent comparator group. Finally, although HRQoL was assessed using validated EORTC instruments, PRO data are inherently subject to response bias, and we could not adjust normative comparisons for comorbidity burden due to a lack of comorbidity-stratified reference data. In addition, the mode of administration (paper vs. electronic) of PRO questionnaires and the limited staff assistance permitted for some patients may have introduced subtle response biases. Indeed, we could not systematically evaluate the impact of digital literacy or differences between electronic and paper completers, particularly in the oldest patients, although all questionnaires were self-completed and staff were instructed not to influence answers. Taken together, these limitations reinforce that this is an exploratory, baseline-only analysis. Future longitudinal PRO-MIND analyses with larger samples will be needed to more formally disentangle disease-, treatment- and age/comorbidity-related determinants of HRQoL. In addition, due to the small sample size, we did not perform formal multivariable or extensive univariable correlations between HRQoL and clinical/biological factors (e.g., disease bulk, ECOG PS, refractory versus relapsed disease, inflammatory markers, number of prior therapies); such analyses are deferred to future PRO-MIND reports when larger samples and outcome data will be available. Given the small sample size, confidence intervals around mean differences and effect sizes are relatively wide, and these estimates may be unstable and sensitive to the inclusion of additional patients. As such, our baseline comparisons should be interpreted as exploratory benchmarks rather than definitive effect-size estimates for all patients with transplant-ineligible R/R DLBCL.

### Clinical Implications and Future Directions

From a clinical perspective, these baseline data argue for systematic HRQoL assessment at the time of the treatment decision in patients with transplant-ineligible R/R DLBCL. In particular, the domains most affected in PRO-MIND—physical, role, cognitive, and social functioning, fatigue, pain, sleep difficulties, appetite loss, and concerns about future health, disease recurrence, and dependency—represent priority targets for supportive and psychosocial interventions even before tafasitamab–lenalidomide is started. Routine use of brief, validated instruments, such as the EORTC QLQ-C30 and QLQ-NHL-HG29, may help clinicians identify patients at greatest risk of functional decline and tailor supportive care accordingly. Because this analysis is restricted to pretreatment HRQoL and does not evaluate tafasitamab–lenalidomide efficacy or safety, it does not provide evidence to support changes in tafasitamab–lenalidomide doses or schedules in specific subgroups, nor to postpone chemoimmunotherapy based on baseline HRQoL alone. Rather, our findings suggest that HRQoL information should be used to inform shared decision-making, to anticipate which patients may require more intensive supportive and psychosocial care, and to interpret subsequent on-treatment HRQoL trajectories in the context of patients’ initial burden of symptoms and functional limitations.

The baseline benchmarks reported here will also be important for interpreting future longitudinal PRO-MIND results. Documenting the starting point of HRQoL and symptom burden in this real-world cohort provides context for evaluating whether tafasitamab–lenalidomide stabilizes, improves, or further compromises HRQoL over time, and for comparing trajectories across clinically relevant subgroups. Ongoing follow-up of the PRO-MIND cohort will therefore complement this cross-sectional analysis by describing on-treatment HRQoL dynamics and their relationship with clinical outcomes.

## 5. Conclusions

In this ad hoc, cross-sectional, baseline-only analysis of the prospective PRO-MIND study, patients with transplant-ineligible R/R DLBCL scheduled to receive tafasitamab–lenalidomide showed pronounced HRQoL impairments compared with age-specific normative values for the Italian general population. The most affected domains were physical, role, cognitive, and social functioning, together with substantial fatigue, pain, insomnia, appetite loss, and pervasive concerns about future health, disease recurrence, and dependency.

These findings provide real-world HRQoL benchmarks prior to tafasitamab–lenalidomide initiation and underscore the need for early, systematic assessment and targeted supportive care focusing on the domains most compromised at baseline. Longitudinal analyses from PRO-MIND will be essential to determine how HRQoL evolves during tafasitamab–lenalidomide treatment and to clarify the impact of treatment on baseline deficits documented in this cohort.

## Figures and Tables

**Table 1:** Baseline demographics and clinical characteristics of patients enrolled in the PRO-MIND study (*N* = 38).

Characteristic	Patients (*N* = 38)
Age, years, median (Q1–Q3)	77.0 (73.0–82.0)
Female sex, *n* (%)	23 (60.5)
Ethnicity, *n* (%)	
White	37 (97.4)
Other	1 (2.6)
Patients with ≥1 comorbidity, *n* (%)	35 (92.1)
Total comorbid events	172
Comorbidities, *n* (%)	
Hypertension	23 (60.5)
Dyslipidemia/hypercholesterolemia	6 (15.8)
Diabetes mellitus	5 (13.2)
Depression	5 (13.2)
Thyroid disorder	4 (10.5)
Atrial fibrillation	4 (10.5)
Osteoporosis	2 (5.3)
Ann Arbor stage, *n* (%)	
Stage II	7/37 (18.9)
Stage III	8/37 (21.6)
Stage IV	22/37 (59.5)
Stage III–IV	30/37 (81.1)
Histology, *n* (%)	
DLBCL NOS	31/37 (83.8)
THRLBCL	1/37 (2.7)
Follicular grade 3b	1/37 (2.7)
Composite lymphoma	4/37 (10.8)
Bulky disease, *n* (%)	8/37 (21.6)
Transplant-ineligible, *n* (%)	37/37 (100)
Best prior response, *n* (%)	
Complete response	15/37 (40.5)
Partial response	17/37 (45.9)
Stable disease	5/37 (13.5)
ECOG PS, *n* (%)	
0	14/30 (46.7)
1	15/30 (50.0)
2	1/30 (3.3)
≥1 prior systemic therapy, *n* (%)	37/38 (97.4)
Prior lines of systemic therapy, *n* (%)	
1	22/37 (59.5)
2	12/37 (32.4)
3	3/37 (8.1)
Received anti-CD20 therapy, *n* (%)	34/37 (91.9)
Anti-CD20 cycles, *n* (%)	
2	1/37 (2.7)
3	2/37 (5.4)
4	2/37 (5.4)
6	26/37 (70.3)
8	6/37 (16.2)
Completed planned cycles, *n* (%)	35/37 (94.6)
Discontinued due to refractory disease, *n* (%)	2/37 (5.4)
First-line regimen, *n* (%)	
R-COMP	22/37 (59.5)
R-CHOP	5/37 (13.5)
Others	5/37 (13.5)
Polatuzumab-based salvage regimen, *n* (%)	2/37 (5.4)
Primary refractory disease, * *n* (%)	25/37 (67.6)

Continuous variables are presented as median (Q1–Q3) and categorical variables as *n* (%). Percentages are calculated using the number of patients with available data as the denominator. Numerators “/37” refer to patients with available data. * Primary refractory disease was defined as lack of response to first-line therapy or disease progression during treatment or within 6 months after completion. Abbreviations: DLBCL, diffuse large B-cell lymphoma; ECOG PS, Eastern Cooperative Oncology Group performance status; NOS, not otherwise specified; Q1–Q3, interquartile range; R-COMP, rituximab-cyclophosphamide-oncovin-non-pegylated liposomal doxorubicin-prednisone; R-CHOP, rituximab-cyclophosphamide-hydroxydaunorubicin-oncovin-prednisone; THRLBCL, T-cell/histiocyte-rich large B-cell lymphoma.

**Table 2:** Prevalence of symptoms and concerns in the PRO-MIND cohort based on the EORTC QLQ-NHL-HG29 (*N* = 38).

Symptom or Concern	*n*	(%)
Worries about future health	32	84.2%
Concerns about disease recurrence	31	81.6%
Worries about becoming dependent	30	78.9%
Lack of energy (fatigue)	27	71.1%
Fear of developing a chronic condition	26	68.4%
Sleep difficulties	24	63.2%
Pain or discomfort	23	60.5%
Problems with memory or concentration	22	57.9%
Difficulties carrying out daily activities	21	55.3%
Social restrictions	20	52.6%
Emotional distress	19	50.0%
Problems at work/place of study	19	50.0%

Data are presented as the number of patients (*n*) and percentage (%) reporting each item at a level of “a little,” “quite a bit,” or “very much.” Abbreviations: EORTC, European Organisation for Research and Treatment of Cancer; HG29, high-grade lymphoma 29 items; NHL, non-Hodgkin lymphoma; QLQ, Quality of Life Questionnaire.

## Data Availability

Incyte Corporation (Wilmington, DE, USA) is committed to data sharing that advances science and medicine while protecting patient privacy. Qualified external scientific researchers may request anonymized datasets owned by Incyte for the purpose of conducting legitimate scientific research. Researchers may request anonymized datasets from any interventional study (except phase 1 studies) for which the product and indication have been approved on or after 1 January 2020, in at least 1 major market (e.g., US, EU, JPN). Data will be available for request after the primary publication or 2 years after the study has ended. Information on Incyte’s clinical trial data sharing policy and instructions for submitting clinical trial data requests are available at: https://www.incyte.com/Portals/0/Assets/Compliance%20and%20Transparency/clinical-trial-data-sharing.pdf?ver=2020-05-21-132838-960 (accessed on 30 October 2025).

## Supplementary Materials

The following supporting information can be downloaded at: https://www.mdpi.com/article/10.3390/diseases13120399/s1, Table S1: EORTC QLQ-C30 mean scores in the PRO-MIND cohort versus age-specific normative values for the Italian general population.
