# Supplementary material for: Health-Related Quality of Life and Symptom Burden in Patients with Diffuse Large B-Cell Lymphoma Before Treatment with Tafasitamab and Lenalidomide: An Ad Hoc Analysis of Italian Real-World Data from the PRO-MIND Study
Source: Diseases. 2025 Dec 15;13(12):399. doi: 10.3390/diseases13120399 (PMC12731899; doi:10.3390/diseases13120399)
Supplement: Supplementary file 1 [file diseases-13-00399-s001.zip › diseases-3957694-supplementary.pdf]

Supplementary Table S1. EORTC QLQ-C30 mean scores in the PRO-MIND cohort versus age-specific normative values for the Italian general population. Mean differences (PRO-MIND – Italian norms), 95% CIs, and effect sizes (Cohen's d). Negative mean differences and Cohen's d for functional scales indicate worse functioning in PRO-MIND versus Italian norms. Positive mean differences and Cohen's d for symptom scales indicate greater symptom burden in PRO-MIND. Abbreviations: C30, Core 30; CI, confidence interval; EORTC, European Organization for Research and Treatment of Cancer; QLQ, Quality of Life Questionnaire; SD, standard deviation.

QLQ-C30 scale	Mean PRO-MIND	SD PRO-MIND	Mean Italian norms	SD Italian norms	Mean difference (PRO-MIND – norms)	95% CI (lower)	95% CI (upper)	Cohen's d
Global health score	59.21	21.60	66.67	18.57	-7.46	-14.81	-0.11	-0.39
Physical functioning	70.00	25.20	82.69	17.75	-12.69	-21.08	-4.30	-0.66
Role functioning	70.17	34.00	86.31	22.01	-16.14	-27.38	-4.90	-0.66
Emotional functioning	74.64	24.20	80.91	17.65	-6.27	-14.35	1.81	-0.33
Cognitive functioning	81.57	24.80	90.09	13.45	-8.52	-16.63	-0.41	-0.54
Social functioning	76.32	24.10	91.51	16.15	-15.19	-23.18	-7.20	-0.86
Fatigue	37.42	27.00	22.58	21.32	14.84	5.75	23.93	0.66
Nausea and vomiting	1.76	10.80	2.74	9.49	-0.98	-4.66	2.70	-0.10
Pain	26.32	23.80	17.62	22.76	8.70	0.49	16.91	0.38
Dyspnea	17.54	25.40	13.49	22.74	4.05	-4.63	12.73	0.17
Insomnia	29.82	31.80	15.93	21.50	13.89	3.34	24.44	0.59
Appetite loss	14.91	24.10	5.54	14.27	9.37	1.45	17.29	0.58
Constipation	12.28	21.10	12.11	21.64	0.17	-7.19	7.53	0.01
Diarrhea	5.26	14.50	5.36	14.13	-0.10	-5.12	4.92	-0.01
Financial difficulties	13.51	24.20	8.14	19.81	5.37	-2.81	13.55	0.26
